# Establishing brain states in neuroimaging data

**DOI:** 10.1371/journal.pcbi.1011571

**Published:** 2023-10-16

**Authors:** Zalina Dezhina, Jonathan Smallwood, Ting Xu, Federico E. Turkheimer, Rosalyn J. Moran, Karl J. Friston, Robert Leech, Erik D. Fagerholm

**Affiliations:** 1 Department of Neuroimaging, King’s College London, United Kingdom; 2 Department of Psychology, Queen’s University, Canada; 3 Child Mind Institute, New York, United States of America; 4 Wellcome Centre for Human Neuroimaging, UCL, United Kingdom; University Medical Center Hamburg-Eppendorf Center for Molecular Neurobiology Hamburg: Universitatsklinikum Hamburg-Eppendorf Zentrum fur Molekulare Neurobiologie Hamburg, GERMANY

## Abstract

The definition of a brain state remains elusive, with varying interpretations across different sub-fields of neuroscience—from the level of wakefulness in anaesthesia, to activity of individual neurons, voltage in EEG, and blood flow in fMRI. This lack of consensus presents a significant challenge to the development of accurate models of neural dynamics. However, at the foundation of dynamical systems theory lies a definition of what constitutes the ’state’ of a system—i.e., a specification of the system’s future. Here, we propose to adopt this definition to establish brain states in neuroimaging timeseries by applying Dynamic Causal Modelling (DCM) to low-dimensional embedding of resting and task condition fMRI data. We find that ~90% of subjects in resting conditions are better described by first-order models, whereas ~55% of subjects in task conditions are better described by second-order models. Our work calls into question the status quo of using first-order equations almost exclusively within computational neuroscience and provides a new way of establishing brain states, as well as their associated phase space representations, in neuroimaging datasets.

## Author summary

There is a deceptively simple question that remains unasked at the heart of computational neuroscience—what exactly is a ’brain state’? This question is motivated by the various and seemingly unrelated definitions of brain states: ranging from the level of wakefulness in anaesthesia, to activity of individual neurons, voltage in EEG, and blood flow in fMRI. There is, however, a precise definition of the state of a dynamical system that often remains overlooked: some piece of information that allow us to say what the system does next. Here, we show that this same definition can be used to quantify the information required to predict the future in neuroimaging timeseries. We demonstrate, with the aid of simulations, that this theoretical framework can be used to extract the characteristic features constituting dynamical system states in a range of scenarios. We then apply the same methodology to fMRI datasets and show that task conditions require more information about a neural system’s history to constitute a brain state, as compared with rest conditions.

## Introduction

The brain is organized into complex networks spanning a vast range of scales, with different networks serving specialized functions [1]. The electrical signals propagating through these networks are organized into distinct patterns of rhythmic activity [2]. When measured by neuroimaging tools, these patterns are referred to as ’brain states’—a term that serves to broadly describe certain time-varying characteristics of the brain [3]. There are a large number of sub-disciplines of neuroscience approaching the question of what constitutes a brain state. A brain state is, for example, referred to as: the membrane potential of individual neurons; [4,5] levels of fluorescence in calcium imaging [6,7]; the concentration of oxygenated blood in fMRI [8,9]; levels of wakefulness with anaesthesia [10,11]; or voltage in EEG [12,13]. This broad range of definitions begs the deceptively simple question: what exactly *is* a brain state?

At the heart of dynamical systems theory, a ’state’ is a sufficient specification of the system’s future [14]. For instance, if we throw a ball into the air then its state consists of two pieces of information—its position and its velocity. This is because knowing where the ball is and how fast it is moving is all we need to determine where it will be next. This is the same premise from which we proceed here in establishing brain states in neuroimaging timeseries.

**Fig 1:**
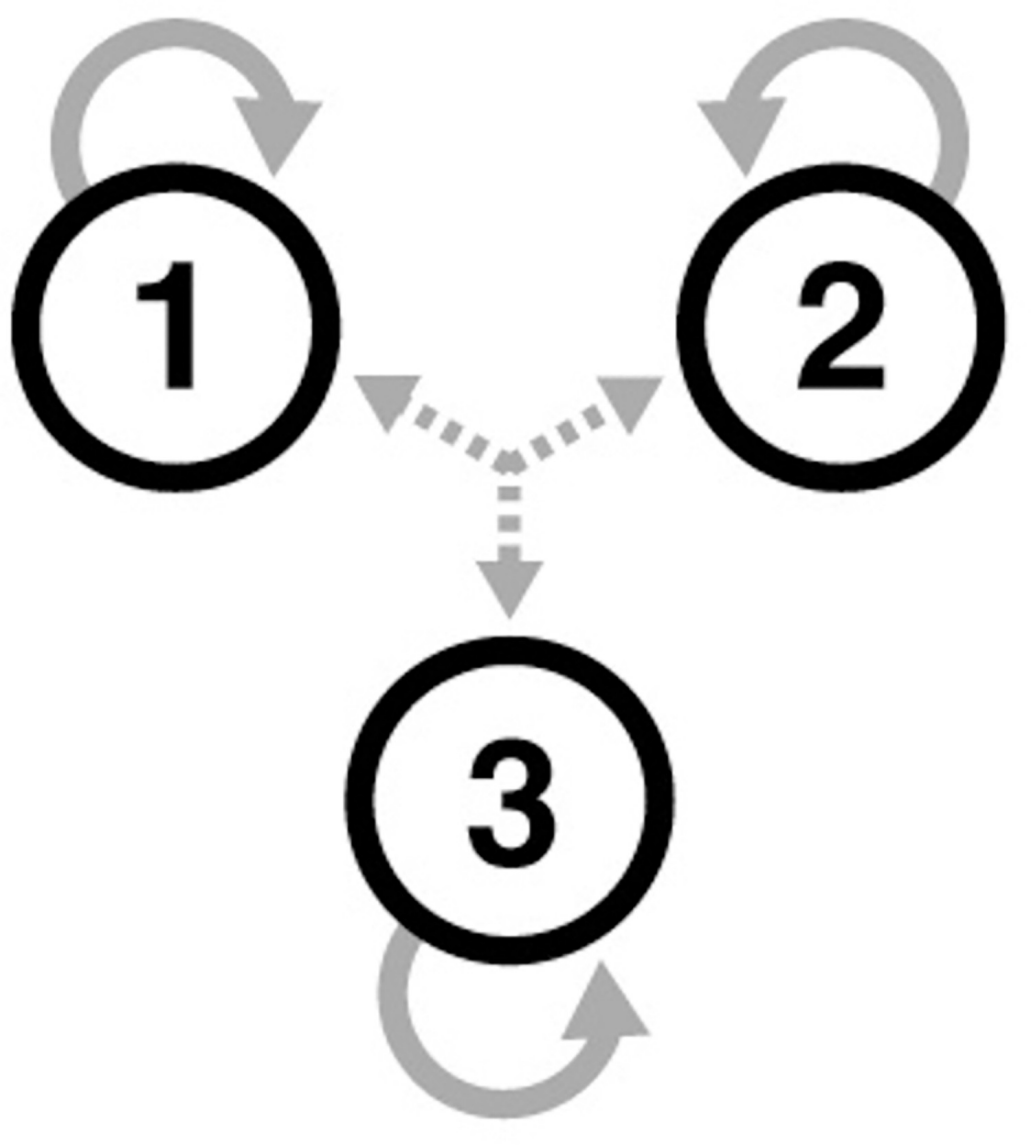
The state of a three-node system in which the regions are coupled only to themselves (i.e., not to one another), as indicated by the looped grey arrows. Each region is acted upon by a driving input, as indicated by the dashed central grey arrows. The future of the system is determined by its own present.

Our approach falls broadly within the remit of other theoretical literature [15–19] in which neural activity is described in terms of a dynamical systems approach. To begin with, we construct a toy model that assumes that whole-brain activity can be represented by three de-coupled regions of interest acted upon by external driving inputs in the presence of noise. This is the situation with which we will deal when representing fMRI data correlated with functional gradients [20]. We first consider the scenario in which each region’s future is determined by its own present (Fig 1).

We can describe the model in Fig 1 in terms of the following system of first-order equations of motion:

$$\left[\begin{array}{c} {\dot{x}}_{1}\\ {\dot{x}}_{2}\\ {\dot{x}}_{3} \end{array}\right]=\left[\begin{array}{ccc} a_{11} & 0 & 0\\ 0 & a_{22} & 0\\ 0 & 0 & a_{33} \end{array}\right]\left[\begin{array}{c} x_{1}\\ x_{2}\\ x_{3} \end{array}\right]+\left[\begin{array}{ccc} c_{11} & 0 & 0\\ 0 & c_{22} & 0\\ 0 & 0 & c_{33} \end{array}\right]\left[\begin{array}{c} v_{1}\\ v_{2}\\ v_{3} \end{array}\right]+\left[\begin{array}{c} \omega_{1}\\ \omega_{2}\\ \omega_{3} \end{array}\right]$$ (1)

where *x*(*t*) are the measured signals and *a* are the internal coupling strengths (the looped grey arrows in Fig 1). For instance, *a*_11_ is the strength with which region 1 is connected to itself. The entire matrix containing the *a* elements acts as a Jacobian [21]—i.e., determining the dynamics of the system in the absence of external perturbation. The *c* elements are the external coupling strengths (the dashed central grey arrows in Fig 1). For instance, *c*_11_ is the strength with which the first driving input affects the first region. The driving inputs are given by *v*(*t*) and the noise terms by *ω*(*t*) [22].

We note the following regarding Eq (1): given known coupling parameters *a* and *c*, each region’s signal one timepoint into the future is determined (via the first derivative) only by the present values of its own measured signal *x*(*t*), the external input *v*(*t*), and the noise *ω*(*t*). In other words, these three pieces of information constitute a ’state’, as they allow us to determine the system’s future. The corresponding phase space—a representation of every possible future of the system—is therefore three-dimensional with axes corresponding to *x*, *v*, and *ω*.

**Fig 2:**
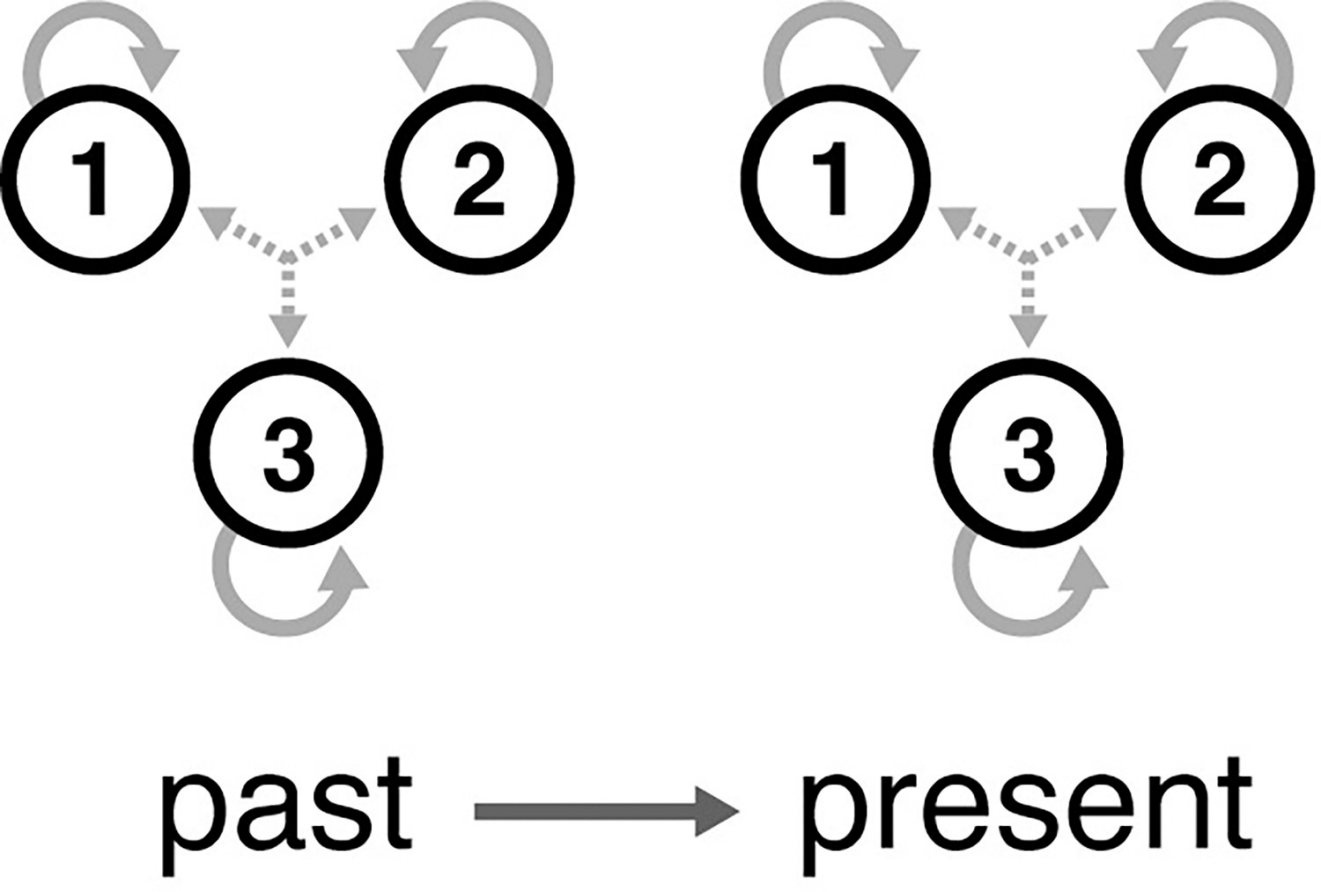
Same as Fig 1, except the state of the system (and hence its future) now depends upon its past as well as its present.

Next we consider the scenario in which each region’s future is determined both by its past as well as by its present (Fig 2).

This model can be described by a system of equations similar in form to Eq (1), except now second-order in time:

$$\left[\begin{array}{c} {\ddot{x}}_{1}\\ {\ddot{x}}_{2}\\ {\ddot{x}}_{3} \end{array}\right]=\left[\begin{array}{ccc} a_{11} & 0 & 0\\ 0 & a_{22} & 0\\ 0 & 0 & a_{33} \end{array}\right]\left[\begin{array}{c} x_{1}\\ x_{2}\\ x_{3} \end{array}\right]+\left[\begin{array}{ccc} c_{11} & 0 & 0\\ 0 & c_{22} & 0\\ 0 & 0 & c_{33} \end{array}\right]\left[\begin{array}{c} v_{1}\\ v_{2}\\ v_{3} \end{array}\right]+\left[\begin{array}{c} \omega_{1}\\ \omega_{2}\\ \omega_{3} \end{array}\right]$$ (2)

We now note that, as opposed to the first-order model in Eq (1), the three pieces of information *x*, *v*, and *ω* only allow us to calculate second derivatives $\ddot{x}$ and thus do not grant access to information about the system one timepoint into the future. Instead, we must add an additional piece of information in the form of the first derivative $\dot{x}$ to solve Eq (2). As such, a ’state’ for each region now consists of four pieces of information $x,\dot{x},v$, and *ω*—constituting the axes of the associated four-dimensional phase space.

Models in computational neuroscience assume—almost without exception—that first-order models (as in Fig 1) are the correct choice, due in part to their computational expediency [23]. However, as expediency sometimes comes at the cost of accuracy, we here question this assumption by directly comparing the first (Fig 1) and second-order (Fig 2) models in their ability to describe neuroimaging timeseries. We present this analysis in two stages. At the first stage, we show that it is possible to correctly identify the information constituting a state for synthetic datasets. These ground-truth simulations are intended to demonstrate proof of principle in the identification of system states via Bayesian model inversion techniques. At the second stage, we apply the same methodology to low-dimensional embedding of rest and task fMRI data obtained from the human connectome project (HCP). In doing so, we show that it is possible to establish brain states via a data-driven approach.

## Methods

### Synthetic datasets

We produce 1000 synthetic timeseries, half of which are generated using a first-order equation of motion (Eq (1), Fig 1) and the other half using a second-order equation of motion (Eq (2), Fig 2). Each timeseries is produced using a model of randomized internal and external connections, as well as a randomized driving input to each of the three regions. We then use Bayesian model inversion to determine whether we can correctly associate each of these synthetic datasets with their associated underlying model (i.e., either first-or second-order in time). In doing so, we set all free parameters (coupling strengths and driving inputs) to Bayesian priors of zero—effectively ’forgetting’ the parameters with which the data were generated.

The model inversion routine in Dynamic Causal Modelling^6^ (DCM) shifts the priors to obtain the most likely values (posteriors) of the free parameters, as well as of the form of the driving input and noise. These routines use Variational Laplace (a generic Bayesian model inversion scheme that assumes a Gaussian form for priors and posteriors) within the Statistical Parametric Mapping (SPM) software to estimate the free parameters, such as the internal and external connectivity strengths. We also estimate the variance of states and hyperparameters via a Laplace approximation in generalised coordinates of motion to estimate the rates of change. This allows for smooth or analytic noise processes [24].

We then calculate the evidence (variational free energy) for each model—i.e., first or second-order—in terms of an approximation to log model evidence, conditioned upon states, parameters and hyperparameters. The model evidence *per se* can be regarded as accuracy minus complexity. Specifically, given data *y*, model structure *m*, hyperparameters *θ*, and approximate posterior density *q*, the accuracy term is given by the log model evidence *logp*(*y*|*θ*, *m*), and the complexity term is given by the Kullback-Leibler divergence KL[q(θ),p(θm)]. The summary statistic (model evidence) of the variational free energy *F* then returns a single number given by the difference of these two terms:

F=accuracy−complexity=〈logp(y|θ,m)〉−KL[q(θ),p(θ|m)]

Posterior model probabilities (’p-values’) are then derived by applying a softmax function $\left(p=\frac{1}{1+e^{\sum F}}\right)$ to the variational free energy.

In other words, the best model is that which accounts for the data accurately but with minimal degrees of freedom (i.e., complexity). This means that, although a first-order model has fewer parameters, it may still provide the greatest overall evidence due to being associated with less complexity than the equivalent model possessing a greater number of parameters.

### Empirical datasets

All rest and task (working memory) fMRI data are taken from the 1200-subject release of the Human Connectome Project (HCP) [25]. These data were collected using a 3T imaging Scanner (Siemens Skyra 3 Tesla MRI scanner, TR = 720 ms, TE = 33 ms, flip angle = 52°, voxel size = 2 mm isotropic, 72 slices, matrix = 104×90, FOV = 208×180 mm, multiband acceleration factor = 8). 100 subjects (ages 22–35) are randomly chosen with 3 subjects excluded due to missing data.

The task-based fMRI data was sourced from the Human Connectome Project S1200 release. All these participants underwent the n-back task, wherein they were exposed to trial blocks with the directive to sequentially monitor the presented images of places, tools, faces, and body parts during each functional magnetic resonance imaging (fMRI) session. Every session was structured to equally divide between 2-back and 0-back working-memory tasks with a 2.5-second instructional cue, succeeded by ten trials each lasting 2.5 seconds for a total of 27.5 seconds. These epochs were interspersed with 15-second baseline intervals [26].

To eliminate global motion and respiratory-related artefacts, noise in the fMRI data was reduced using the ICA-FIX based pipeline. ICA-FIX (FMRIB’s ICA-based Xnoiseifier) is a pre-processing pipeline used for cleaning fMRI data by removing noise-related components that can interfere with the analysis. This pipeline combines two different methods of data preprocessing: independent component analysis (ICA) and FMRIB’s Automated Removal of Motion Artefacts (FMRIB-AROMA) [27,28].

A group-average functional connectivity matrix was obtained from a subset of 1200 MSMAII-registered individuals acquired through the HCP. For each subject, a functional connectivity matrix was calculated using the correlation coefficient across four FIX-ICA de-noised (i.e., the same procedure as for all timeseries analysed) 15-min resting-state fMRI scans. The latter were parcellated according to a 100-region parcellation atlas [29]. We then projected this matrix into a low-dimensional space using the diffusion embedded approach [30] within the BrainSpace toolbox [31]. Specifically, we created three dimensions of brain variation determined from the application of decomposition techniques to resting state data. These dimensions of brain variation, often described as ’gradients’, allow for patterns of similarity in brain activity to be visualized across the cortex. Note that we restrict the models (Figs 1 and 2) to contain self-connections only to reflect the orthogonalization imposed by the eigendecomposition implicit in diffusion map embedding.

Gradients are ranked based on the variance accounted for by each component, which in turn allows for brain regions to be sorted in terms of dynamic similarity. Brain regions at one end of a given gradient have similar fluctuations in activity over time, and collectively show less similarity to the regions located at the other end [32]. The advantage of this method, in contrast with other parcellation approaches, is that one does not need to define any cortical boundaries *a priori*. Instead, gradients are calculated in an entirely data-driven way, which allows for new methods of analysis with regard to gradually varying neuronal properties [20,33].

Prior studies have used this approach to understand common changes in neural activity and experience [34], as well as to understand how dynamic states are organized at rest and how they relate to trait variance in experiences and affective processes [35]. The first (principal) gradient tracks a functional hierarchy from primary sensory processing to higher-order functions such as social cognition [20]. The second gradient separates visual regions at one end from somatomotor and auditory regions at the opposite end [36]. A prominent axis of functional connectivity variance emerges, delineating two sets of regions: unimodal and transmodal. Unimodal regions, predominantly situated in the somatomotor cortex, are specialized neural areas dedicated to processing information from a singular sensory modality. For instance, the primary visual cortex exclusively processes visual stimuli, exemplifying the specificity of unimodal regions. Conversely, transmodal regions, anchored around the default network and the superior frontal gyrus, undertake a more integrative role. Rather than being confined to a single sensory domain, these regions integrate information across multiple sensory modalities, facilitating higher-order cognitive functions. A simple example of this is the default mode network, which becomes notably active during introspective tasks, such as self-reflection or daydreaming [37]. The third gradient spans the default mode at one end and frontal parietal networks within the association cortex at the other [38]. Note that we also examine the fourth gradient to test consistency of results. Averaged across subjects and scanning conditions, gradients one through four explain a total of 47%, 26%, 16%, and 9% of the variance in the data.

We correlate every subject’s data with these gradients to determine the extent to which each time point is related to each gradient. Using the same approach as for the synthetic data, we then use Bayesian model inversion to determine whether each dataset is better described by first or second-order dynamics. Note that the inversion scheme uses a simplified form of stochastic DCM (i.e., ignoring hemodynamics); namely, fitting observed timeseries to models in which the motion of latent or hidden states are subject to analytic noise. This means that one has to estimate both the states and parameters of the ensuing state space models, as well as the precision of state and observation noise. This allows us to precisely define the features that constitute a neural state on a subject-specific level in a data-driven way.

Finally, we circularly shift each of the 100 regions’ time courses by different random amounts prior to correlating with the three gradients. This ensures that, although the power spectra and intra-regional temporal dependences are preserved, the inter-regional temporal dependences are destroyed—thereby creating a fitting null model.

## Results

All results can be reproduced using the accompanying MATLAB code.

### Synthetic data

**Fig 3:**
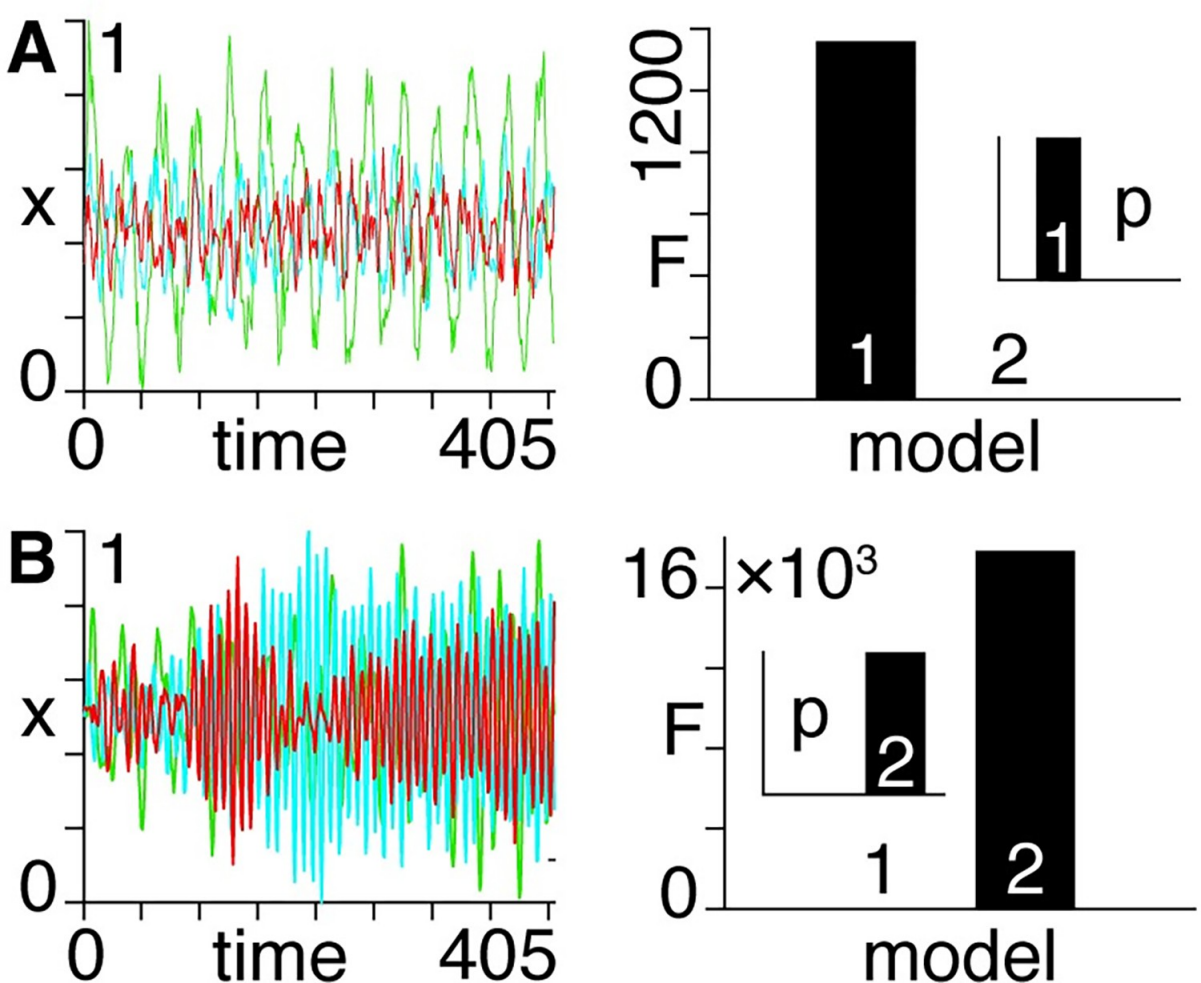
**A)** Left: first-order synthetic timeseries with normalized dependent variable (x). Right: Approximate log model evidence (variational free energy, ’F’) and associated probabilities (’p’, inset) following Bayesian model inversion, demonstrating that this timeseries is correctly associated with a first-order model. **B)** Same as A), except the timeseries on the left is second-order, as identified by the associated model comparison on the right.

We produce synthetic timeseries that are generated using either first or second-order equations of motion with randomized model parameters and driving inputs. We then demonstrate that Bayesian model inversion can be used to correctly associate the first-order dataset with a first-order model (Fig 3A). Similarly, we show that we can correctly associate the second-order dataset with a second-order model (Fig 3B).

We then repeat the procedure in Fig 3 for 1000 randomized timeseries (first 10 shown in S1 Fig)—where 100% are correctly associated with the order with which they were generated.

### Empirical data

**Fig 4:**
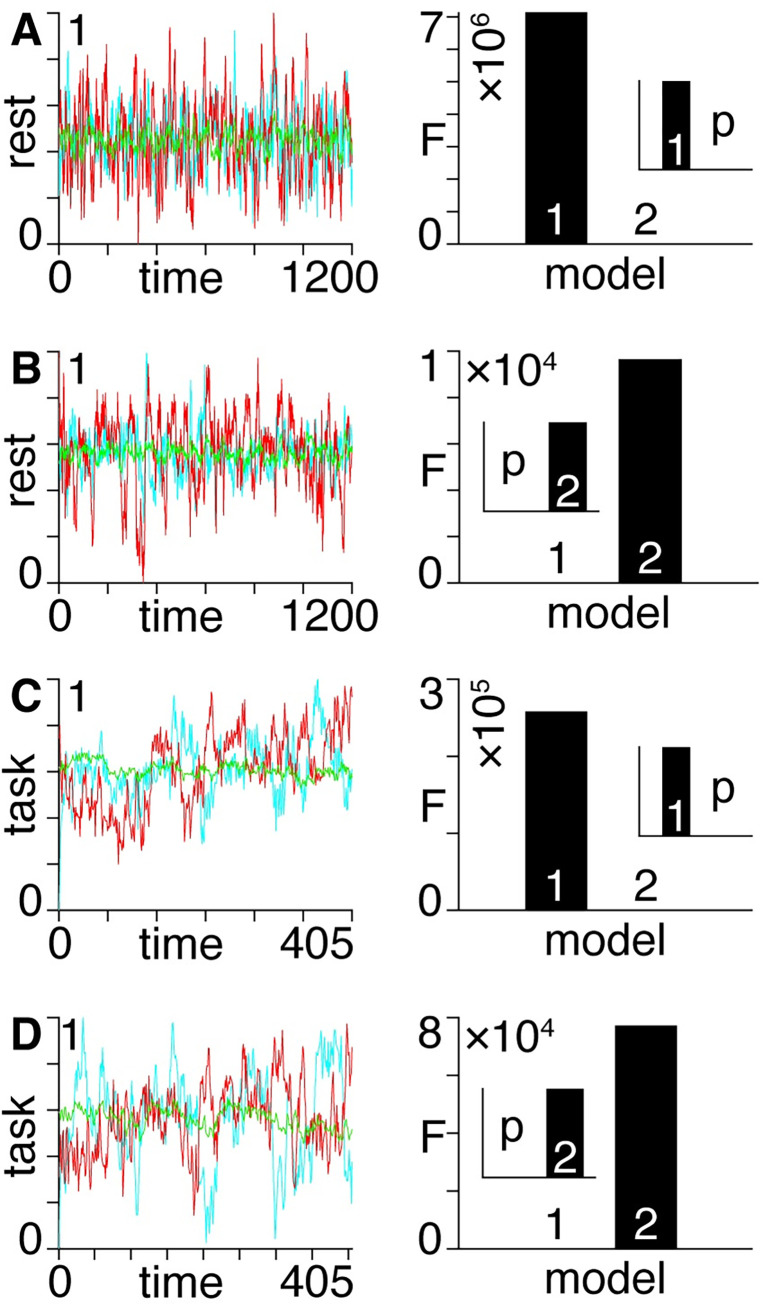
Left: gradient-based timeseries with normalized dependent variables for single subjects. Right: approximate log model evidence (variational free energy, ’F’) and associated probabilities (’p’, inset) for first and second-order dynamics following Bayesian model inversion. **A)** An example of rest condition better described by a first-order model. **B)** An example of rest condition better described by a second-order model. **C)** An example of task condition better described by a first-order model. **D)** An example of task condition better described by a second-order model.

Applying the same Bayesian model inversion technique used for the synthetic datasets we then determine, on an individual-subject level, whether neural dynamics in gradient-based rest and task condition fMRI timeseries are better explained by first or by second-order models. We display example timeseries for subjects in which the rest condition is better described by first (Fig 4A) or by second (Fig 4B) order timeseries. Similarly, we display example timeseries in which the task condition is better described by first (Fig 4C) or by second (Fig 4D) order timeseries.

**Fig 5:**
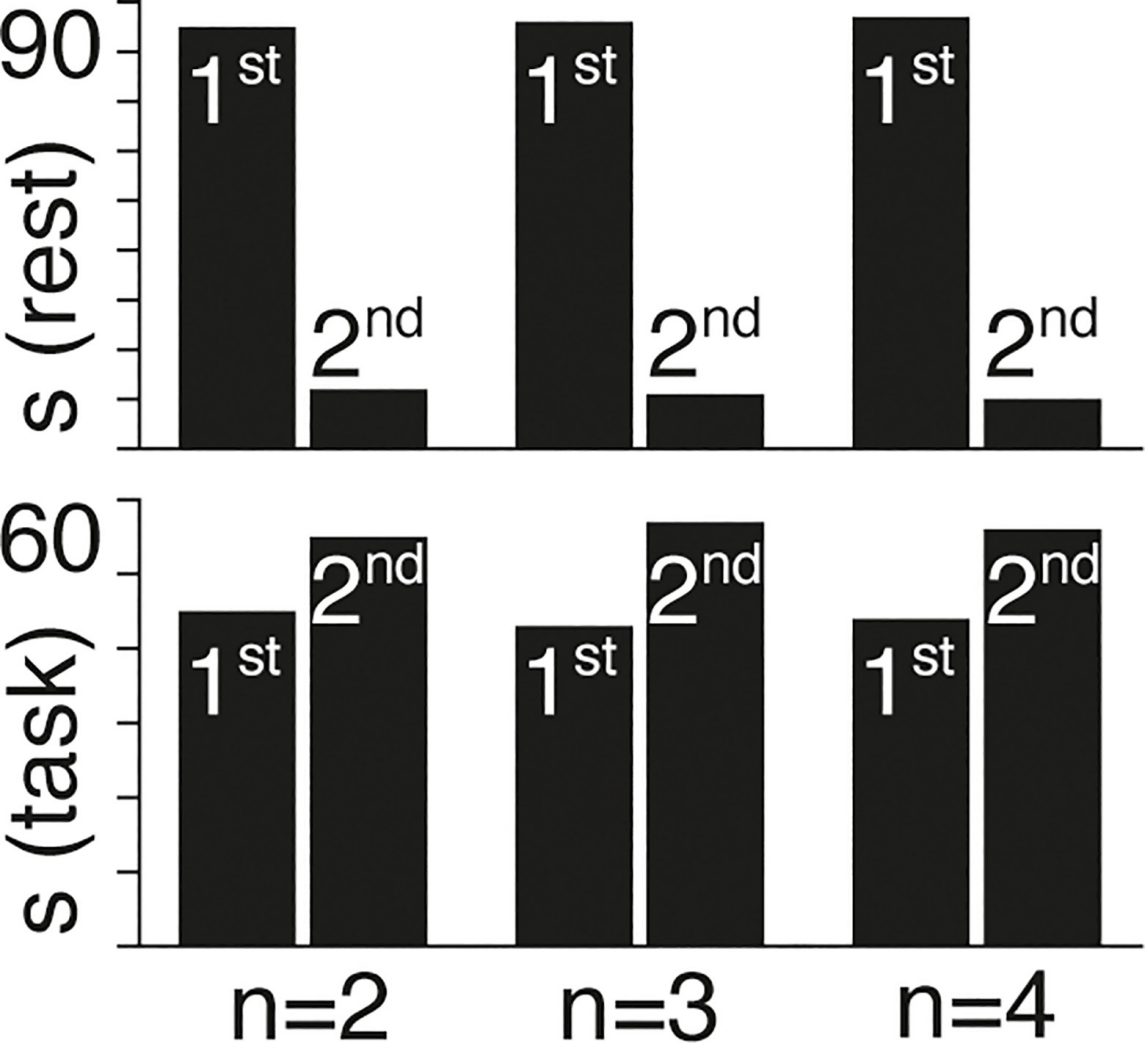
The proportion of subjects (s) that are better described by 1^st^ and 2^nd^ order models in rest (top) and task (bottom) conditions for *n* = 2, *n* = 3, and *n* = 4 gradients.

We summarize the results across all subjects, showing the number of subjects better described by first or by second-order models in rest and task conditions for *n* = 2, *n* = 3, and *n* = 4 gradients (Fig 5). We find that consistently ~ 90% of subjects in the rest condition are better described by first-order models, whereas a slim majority (~ 55%) of subjects in the task condition are better described by second-order models.

We find that there is no difference between the task performance (median reaction times) of the subjects that are better modelled by first vs. second-order dynamics.

**Fig 6:**
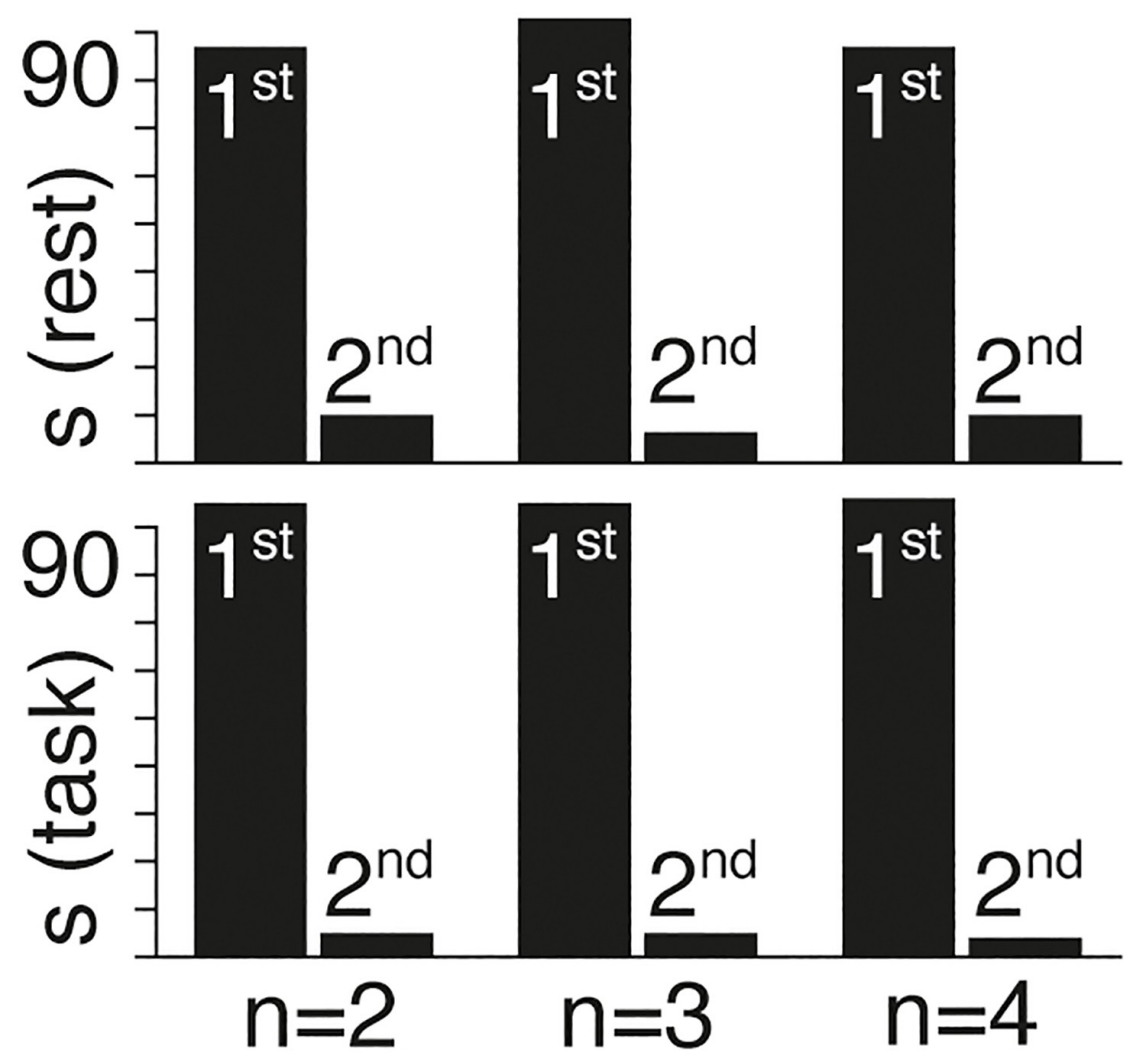
The proportion of subjects (s) that are better described by 1^st^ and 2^nd^ order models in rest (top) and task (bottom) conditions for *n* = 2, *n* = 3, and *n* = 4 gradients, for circularly shifted timeseries.

Upon circularly shifting the timeseries of each region by random amounts prior to gradient correlation, we find that the large majority of subjects in both rest and task conditions are better described by first order models (Fig 6).

**Fig 7:**
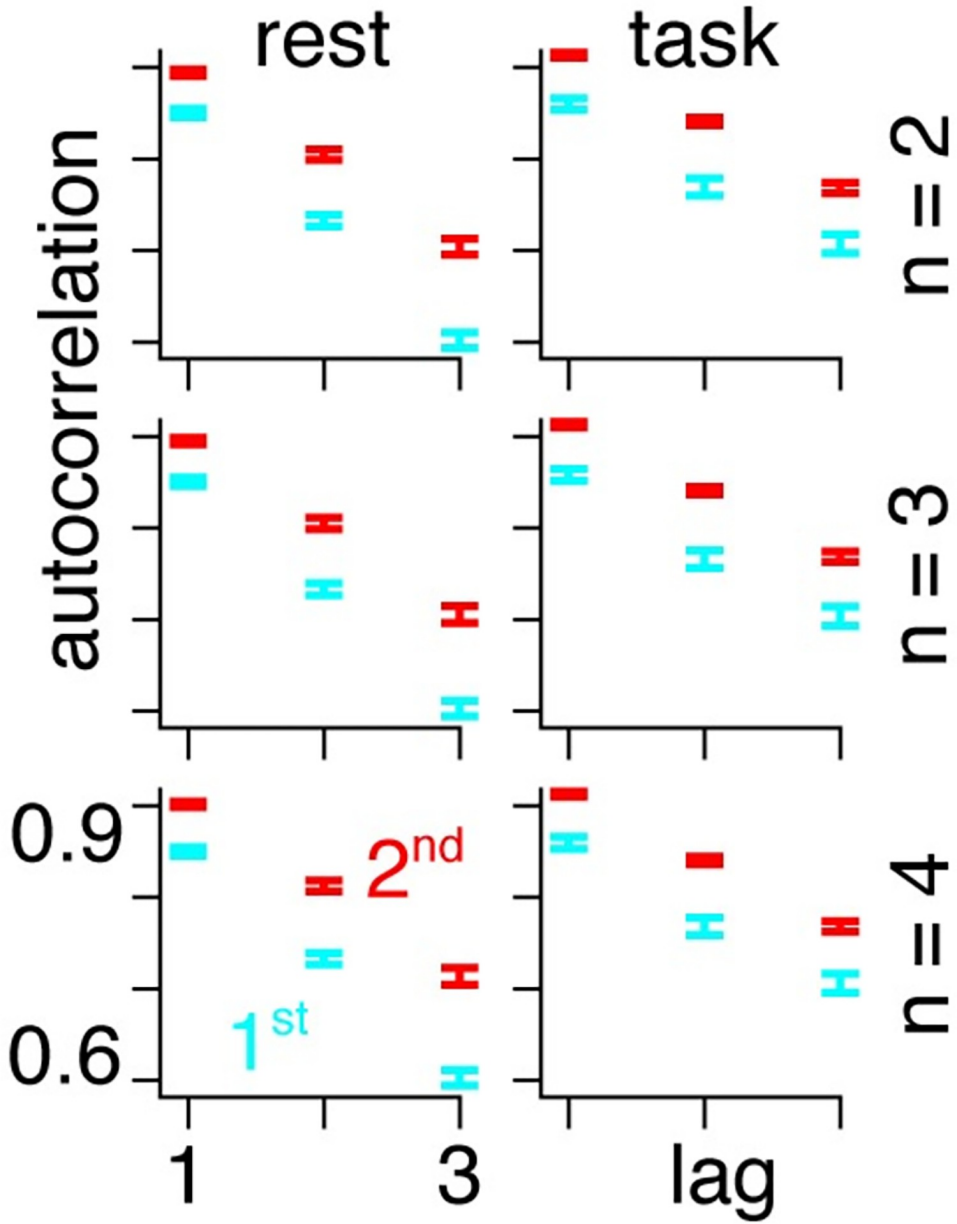
Temporal autocorrelation (y-axis) as a function of the lag (x-axis) for rest (left column) and task (right column) for n = 2 (first row), n = 3 (middle row) and n = 4 (bottom row) gradients. The results show that the subjects that are better described by second-order models (red) have consistently higher auto-correlations than the subjects that are better described by first-order models (blue).

To place these results in a more common framework, we calculate the temporal autocorrelation in the subjects that are better described by second-order models and find that they are consistently higher than in the subjects that are better described by first-order models across scanning conditions and numbers of gradients (Fig 7).

## Discussion

Our study demonstrates that it is possible to establish the information constituting the state of a neural system using a theoretical framework employed in dynamical systems theory. We first provided proof of principle by generating synthetic datasets and by using DCM to recover the associated model parameters—limited by the underlying assumption of linearization (see Eqs (1) and (2)). Next, we extended this analysis to neuroimaging data using dimensions of variation (gradients) derived from a decomposition of temporal variations in resting state data from the HCP. We used these gradients to generate a set of co-ordinates for the timeseries and used DCM to determine whether the datasets are better described by first or by second-order equations of motion.

We found that a clear majority of subjects in rest conditions are better described by first-order models, whereas a narrow (but consistent) majority of subjects are better described by second-order models. This indicates that the system’s history, as encoded in the second-order model, is of greater relevance in predicting the future of a task-condition timeseries.

Some of the results could be related to the increased temporal autocorrelation in the timeseries that are better modelled by second vs. first-order equations of motion. Note that, due to the onset of Ostrogradsky instabilities, we restrict the differential equations used in our analysis to be maximally of order two, even though higher orders would be necessary to accommodate the effect of longer temporal delays [39,40]. On the other hand, the effects of such temporal delays are less prominent in fMRI data due to the associated low temporal resolution.

The use of second-order models in computational neuroscience can also be motivated by the evidence that the brain exhibits temporal auto-correlation [41]. Furthermore, temporal auto-correlation is known to be organised according to the unimodal-transmodal axis (principal gradient), being greater at the transmodal than at the unimodal end [42,43]. As such, one may expect the correlation between BOLD signals and the principal gradient to vary more because of changes at the unimodal end, than at the transmodal end. This in turn may suggest that correlations with gradients are a potential confound with respect to spatial and temporal characteristics.

The phase space of a dynamical system possesses the dimensionality of its attracting set (i.e., the set of points in phase space to which the system converges). This dimensionality is the focus of many procedures in dynamical systems theory [44–46]. For example, the Grassberger-Procaccia algorithm [47] attempts to identify this minimum number by examining the correlation dimension. Often, these kinds of schemes rest upon Takens’ embedding theorem—a delay embedding theorem that furnishes the conditions under which a (chaotic) dynamical system can be reconstructed from a sequence of observations of its state. As opposed to the noise-free assumption underlying Takens’ theorem, we applied Bayesian model selection in which the (dynamic causal) models with increasing numbers of differential equations allow for noise that is intrinsic to the data generating process in addition to observation noise.

The ability to represent the totality of a system’s states in the form of a phase space unlocks several novel perspectives with regard to the study of the brain. For instance, in a discrete system the entropy is defined in terms of the average log probability of observing a system in its various states. On the other hand, the continuous extension of this discrete form of entropy relates to the volume in phase space, expressed in terms of a probability density function of continuous random variables. Access to the phase space therefore allows for a formally derived measure of the hidden information (entropy) contained within the system [48].

Furthermore, having constructed a data-driven phase space via timeseries as shown here, we are in a position to directly assess associated predictability by measuring the rate of divergence (via a positive Lyapunov exponent) of trajectories in phase space [49,50]. However, it is not possible to resolve infinitesimally close points in phase space due to limits in resolution or lack of knowledge regarding initial conditions. In reality we must treat any representation as having been ‘smeared’ (coarse-grained) across finite-size sub-volumes within phase space. Following this process, points that are closer to one another than the size of a single sub-volume become indistinguishable from one another [51]. Having constructed data-specific phase space representations, we can use the methodology presented here to test ranges of sub-volume sizes to assess appropriate resolution limits across neuroimaging modalities.

The reach of our existing implementation goes beyond the study of cognitive and systems neuroscience [35]. Different scenarios and data modalities will result in different phase spaces and as such, we intend for our work to highlight a principled framework for defining system states in a data-driven way. It should be noted, however, that the models focus on the information content required to predict the future of a region of interest, as opposed to the origin of said information content. This means that potential parallel processing [52,53] taking place within a single region or network is not accounted for by our model.

Importantly, since prior studies have highlighted the utility of state representations as a tool for identifying individual variation [34], our approach could be extended to more clearly show how brain activity patterns relate to variation in cognitive and affective features of behaviour. For instance, Reid et al. discuss the challenges involved in describing brain network interactions through functional connectivity analysis—a better understanding of which can lead to more accurate predictions of future patterns of neural activity [54]. Their emphasis on understanding causal interactions among neural entities aligns with our approach of defining brain states in terms of sufficient information in predicting future timepoints.

On the scale of neural populations, brain states are sometimes characterized as a global neural assembly with specific dynamics (e.g., ranges of firing rates, single neuron variability, synchronization across neurons) affected by neuromodulation [55]. Our approach can be used to build on this premise in a way that is applicable across various methods of measuring brain activity. It is our hope that this work provides a formalisation of neural dynamics that will help to link this heterogeneous research landscape.

## Supporting information

S1 Fig The first 10 of 1000 timeseries with normalized dependent variables (x), generated using randomized model parameters and driving inputs. Each row contains, from left to right: a timeseries produced using a first-order equation of motion; the model evidence ’F’ for whether this ground-truth first-order timeseries was produced with first or second-order dynamics; a timeseries produced using a second-order equation of motion; and the model evidence ’F’ for whether this ground-truth second-order timeseries was produced with first or second-order dynamics.
(TIF)
