# Supplementary figures and images for: Establishing brain states in neuroimaging data
Source: PLoS Comput Biol. 2023 Oct 16;19(10):e1011571. doi: 10.1371/journal.pcbi.1011571 (PMC10602380; doi:10.1371/journal.pcbi.1011571)

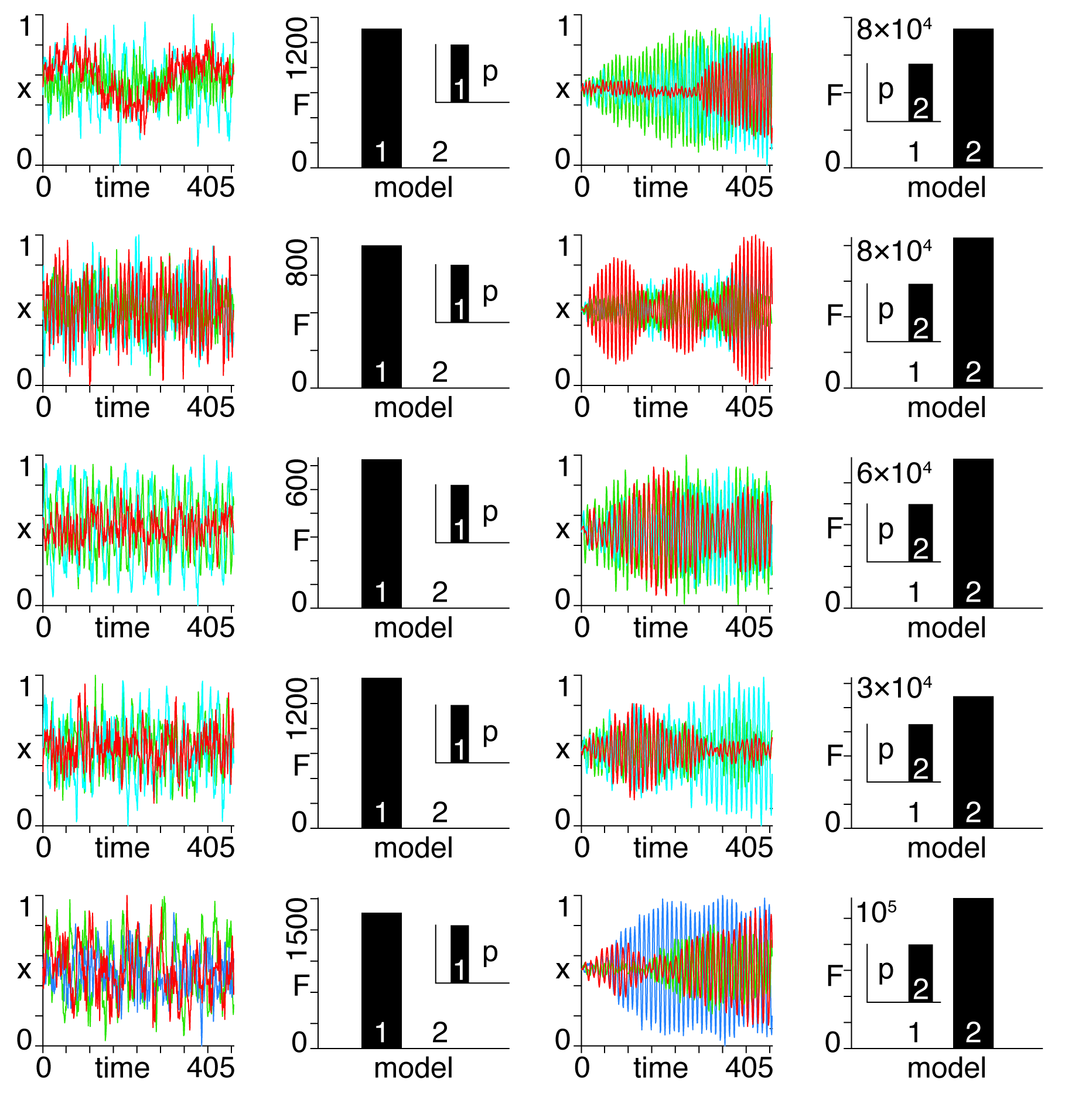

Supplement: S1 Fig — Each row contains, from left to right: a timeseries produced using a first-order equation of motion; the model evidence ’F’ for whether this ground-truth first-order timeseries was produced with first or second-order dynamics; a timeseries produced using a second-order equation of motion; and the model evidence ’F’ for whether this ground-truth second-order timeseries was produced with first or second-order dynamics. (TIF) [file pcbi.1011571.s001.tif]
